# Allogeneic administration of human umbilical cord-derived mesenchymal stem/stromal cells for bronchopulmonary dysplasia: preliminary outcomes in four Vietnamese infants

**DOI:** 10.1186/s12967-020-02568-6

**Published:** 2020-10-20

**Authors:** Liem Thanh Nguyen, Thai T. H. Trieu, Hue T. H. Bui, Van T. Hoang, Anh T. T. Nguyen, Nhung T. H. Trinh, Kien T. Nguyen, Duc M. Hoang

**Affiliations:** 1 Vinmec Research Institute of Stem Cell and Gene Technology, Vinmec Healthcare System, Hanoi, Vietnam; 2 Vinmec International Hospital—Times City, Vinmec Healthcare System, Hanoi, Vietnam; 3 Vinmec Hi-Tech Center, Times City, Vinmec Healthcare System, Hanoi, Vietnam; 4 Cellular Manufacturing Department, Vinmec Research Institute of Stem Cell and Gene Technology, 458 Minh Khai, Hai Ba Trung Ward, Hanoi, Vietnam

**Keywords:** Bronchopulmonary dysplasia, Umbilical cord tissue, Allogeneic mesenchymal stem cell administration

## Abstract

**Background:**

Bronchopulmonary dysplasia (BPD) is a severe condition in premature infants that compromises lung function and necessitates oxygen support. Despite major improvements in perinatal care minimizing the devastating effects, BPD remains the most frequent complication of extreme preterm birth. Our study reports the safety of the allogeneic administration of umbilical cord-derived mesenchymal stem/stromal cells (allo-UC-MSCs) and the progression of lung development in four infants with established BPD.

**Methods:**

UC tissue was collected from a healthy donor, followed by propagation at the Stem Cell Core Facility at Vinmec Research Institute of Stem Cell and Gene Technology. UC-MSC culture was conducted under xeno- and serum-free conditions. Four patients with established BPD were enrolled in this study between May 25, 2018, and December 31, 2018. All four patients received two intravenous doses of allo-UC-MSCs (1 million cells/kg patient body weight (PBW) per dose) with an intervening interval of 7 days. Safety and patient conditions were evaluated during hospitalization and at 7 days and 1, 6 and 12 months postdischarge.

**Results:**

No intervention-associated severe adverse events or prespecified adverse events were observed in the four patients throughout the study period. At the time of this report, all patients had recovered from BPD and were weaned off of oxygen support. Chest X-rays and CT scans confirmed the progressive reductions in fibrosis.

**Conclusions:**

Allo-UC-MSC administration is safe in preterm infants with established BPD.

*Trial registration *This preliminary study was approved by the Vinmec International Hospital Ethics Board (approval number: 88/2019/QĐ-VMEC; retrospectively registered March 12, 2019).

## Background

First discovered in 1967, bronchopulmonary dysplasia (BPD) has since emerged as the most prevalent chronic lung disorder in premature infants, resulting in reductions in alveolarization, vascular growth and overall lung function [1]. According to the National Institute of Child Health and Human Development (NICHD), BPD is defined as a persistent parenchymal lung disease in preterm infants (< 32 weeks gestational age) with radiographic confirmation, and at 36 weeks postmenstrual age, BPD requires oxygen support for more than 3 consecutive days to maintain arterial oxygen saturation in the 90–95% range [2]. The pathological hallmarks of BPD involve disruption of lung development, impaired alveolarization and interstitial fibrosis due to antenatal (intrauterine growth restriction, maternal smoking) and/or postnatal risk factors (mechanical ventilation, oxygen toxicity, and infection) [3, 4]. BPD commonly occurs in preterm infants who weigh less than 1000 g, are born at 24–26 weeks of gestation and require prolonged mechanical ventilation and oxygen support [5]. Infants at less than 30 weeks gestational age are at a particularly high risk for immature respiratory system development and suffer from detrimental long-term outcomes, including high morbidity and mortality rates. In the last 50 years, advances in neonatal medicine, including the discovery of neonatal steroid treatments, which were proven to be associated with glucocorticoid-related brain injury as major side effect [6–8], surfactants [9–11], gentle ventilation treatments [12, 13], and effective noninvasive ventilation devices, have significantly improved the clinical outcomes in premature newborns with BPD. However, the rates of complications and mortality are still high among infants with BPD [14].

Recently, MSC therapy was used to treat BPD in an animal model. Proof-of-concept experiments in neonatal BPD rodent models demonstrated that the injection of bone marrow mesenchymal stem cells (BM-MSCs) via either the intravenous (IV) or intratracheal route had lung-protective functions, including reducing lung inflammation and pulmonary hypertension and reforming the alveolar structure, subsequently improving the survival rate [15–18]. Furthermore, a single dose of human UC-MSCs administered intratracheally prevented and rescued neonatal rats from hyperoxia-induced lung damage [19]. In humans, Ahn and colleagues conducted the first phase I clinical trial using umbilical cord blood-derived MSC (UCB-MSC) administration to prevent the manifestation of BPD in premature infants in 2014. Their results confirmed that UCB-MSC administration was safe in premature infants at risk of BPD development [20]. In 2017, our group reported the first patient with established BPD treated successfully with autologous bone marrow mononuclear cells [21]. However, obtaining bone marrow from established BPD newborns in critical condition is challenging and carries a major risk of pulmonary complications. Therefore, this study was performed to evaluate the safety of allogeneic administration of UC-MSCs based on their immunoprivilege features and eliminate the need for bone marrow aspiration in infants with established BPD. We hypothesized that allo-UC-MSC administration is safe for established BPD infants.

## Methods

### Ethics

This study was approved by the Scientific and Ethics Committee of Vinmec International Hospital (approval number: 88/2019/QĐ-VMEC). Written consent was obtained from both the umbilical cord donor and the patients’ parents.

### Donor screening criteria for UC tissue

Healthy women with an uncomplicated, at term pregnancy underwent serological testing, including tests for HIV, cytomegalovirus (CMV), Epstein-Barr virus (EBV), hepatitis A virus (HAV), hepatitis B virus (HBV), hepatitis C virus (HCV), syphilis, and chlamydia, at 38 weeks of pregnancy. The umbilical cord tissues were collected at delivery and transferred to the laboratory for further processing. A single UC sample was selected for isolation of therapeutic MSCs.

### Allo-UC-MSC preparation

A single eligible UC sample was chosen for processing at the Stem Cell Core Facility at the Vinmec Research Institute of Stem Cell and Gene Technology under ISO 14644-1 (certification number: CR61119-1). Culture reagents were purchased from Thermo Fisher Scientific (https://www.thermofisher.com/) and Pan Biotech (serum-free PowerStem MSC1 culture media, P04-77355 K, hereafter called MSC culture media) unless stated otherwise. hUC-MSC cultures were conducted under xeno- and serum-free conditions at 37 °C in a humidified incubator containing 5% CO_2_. The medium was changed every 3 days until the culture reached 80% confluence, followed by passaging using CTS™ TrypLE™ Select (A1285901). UC-MSCs were cryopreserved at passage (P) 3 in the serum- and xeno-free defined reagent CryoStor® CS10 (Stem Cell Technology, Canada) in liquid nitrogen (gas phase) in an automated Brooks System (Brooks Life Science, USA) for long-term storage.

To prepare UC-MSCs for therapy, aliquots of hUC-MSCs at P3 were thawed in CTS™ CELLstart™ substrate-coated flasks and cultured using TryPLE passaging; under these conditions, hUC-MSCs were routinely passaged by incubation with 1X CTS™ TrypLE™ Select for 4 min at 37 °C to liberate single cells or, preferably, small clumps of cells and subcultured for further expansion at a seeding density of 5000 cells/cm^2^. At P5, the cells were harvested using TryPLE as described above if no bacteria, fungi, mycoplasma, or endotoxins were detected and suspended in 10 ml of NaCl 0.9% (Braun, USA) at a final dose of 1 × 10^6^ cells/kg patient body weight (PBW) prior to delivery to the administration ward.

### Product release criteria

To generate and release the final product, hUC-MSCs at P5 were freshly harvested and subjected to a quality control process including (1) cell enumeration, (2) cell viability measurement (> 85%), (3) hMSC marker analysis by a Navios flow cytometer system (Beckman Counter) using a human BD Mesenchymal Stem Kit (562,245, BD Biosciences), (4) microbiological tests for sterility, (5) a test for mycoplasma, (6) determination of the endotoxin level, (7) karyotyping, (8) a CFU assay, and (9) trilineage differentiation using StemPro™ Adipogenesis (A1007001), StemPro™ Chondrogenesis (A1007101), and StemPro™ Osteogenesis Differentiation (A1007201) kits according to the manufacturers’ protocols. Oil Red O, Alicante Blue, and Alizarin Red S were used to specifically stain adipocytes, chondrocytes, and osteocytes, respectively.

### Patient enrollment

#### Inclusion criteria

1. Patients diagnosed with BPD (premature infant with a gestational age less than 32 weeks and required oxygen support (> 21%) for at least 28 days) according to NICHD guidelines [2].
2. Infants who underwent conventional BPD treatment (including neonatal steroid treatments, surfactants, and gentle ventilation support) without conditional improvement and remained dependent on oxygen support (FiO_2_ > 21%).
3. Infants aged between 0 and 1 years old.

#### Exclusion criteria

1. Patients with complex heart abnormalities, congenital diaphragmatic hernia.
2. Patients with other severe conditions (active pulmonary bleeding, evidence of active infections, septic shock, unstable pulmonary hemorrhage).

### Mode of cell administration

All four patients received two administrations of allo-UC-MSCs at a dose of 1 million cells/kg PBW via the IV route with a 7-day intervening interval. On the day of infusion, harvested cells (P5) at the targeted dose were prepared in 10 mL of 0.9% NaCl (Braun, USA) as described above and delivered to the administration ward for IV infusion at a rate of 20 mL/hour.

### Outcome measures

To assess safety, any major or minor adverse events during the MSCs infusion (72 h) and during the 7 days after infusion were monitored. Body temperature, blood pressure, respiratory rate, heart rate, and SpO_2_ were recorded regularly. All four patients were requested to attend re-examination at the hospital at 7 days, 1, 6 and 12 months after discharge. Each visit involved a full clinical assessment, including height and body weight measurements. All medications, home oxygen therapies, and rehospitalizations since the last visit were documented. SpO_2_ and arterial blood gas (ABG) analysis were examined at baseline and at each visit. Chest X-rays and CT scans were performed prior to intervention at the 6-month (CT scan) and 12-month (chest X-ray) visits (Table 1).

**Table 1:** General characterization of patients enrolled in the study

Characteristics	Patient 1	Patient 2	Patient 3	Patient 4
DOB	25/05/2018	25/05/2018	01/08/2018	07/07/2018
Gestational age at birth, weeks	24 (+ 5 days)	24 (+ 5 days)	34	28
Birth weight (grams)	720	650	2400	1400
Sex	Female	Female	Male	Female
Prenatal steroids used	Yes	Yes	No	No
Pulmonary hypertension	Yes	Yes	No	Yes
Mechanical ventilation duration before transplantation	3.5 months	4.5 months	1 month	3 months
PaCO_2_ level (mmhg) before transplantation	37.9	68	59	38.6
HCO_3_– (mmHg) before transplantation	29.1	41.3	47.2	29.5
Oxygen support before transplantation	Nasal cannula 0.5 l/min	Nasal cannula 1.0 l/min	Nasal cannula 1.0 l/min	Nasal cannula 0.5 l/min
PaCO_2_ at 6 months	38	32.8	28.6	39.8
SpO_2_ (%) FiO_2_ (21%) before transplantation	75	91	70	91
SpO_2_ at 6 months	100	95	99	97
SpO_2_ at 12 months	100	100	100	97
Postnatal age at UC-MSC administration, days	144	151	173	160
Weight at UC-MSC administration (grams)	3600	4000	5400	3800
Duration from birth to discharge (days)	161	161	183	173
Duration from transplantation to independence from oxygen support	3 days after the 2nd transplantation	2 months	2 months	4 days after the first transplantation
Chest X-ray before transplantation	Diffuse fibrosis, atelectasis, diffuse haziness	Diffuse fibrosis, atelectasis, diffuse haziness	Air trapping, diffuse fibrosis, atelectasis, diffuse haziness	Air trapping, diffuse fibrosis, atelectasis, diffuse haziness
Chest X-ray 12 months after transplantation	Reduction in fibrosis	Reduction in fibrosis	Normal	Normal

### Statistical analysis

The data were analyzed using one-way ANOVA with Prism GraphPad software unless otherwise stated. ANOVA was performed to compare the means of the four patients as indicated in the test. Statistical significance was defined as P < 0.05 unless otherwise indicated.

## Results

### hUC-MSC characterization

**Fig. 1:**
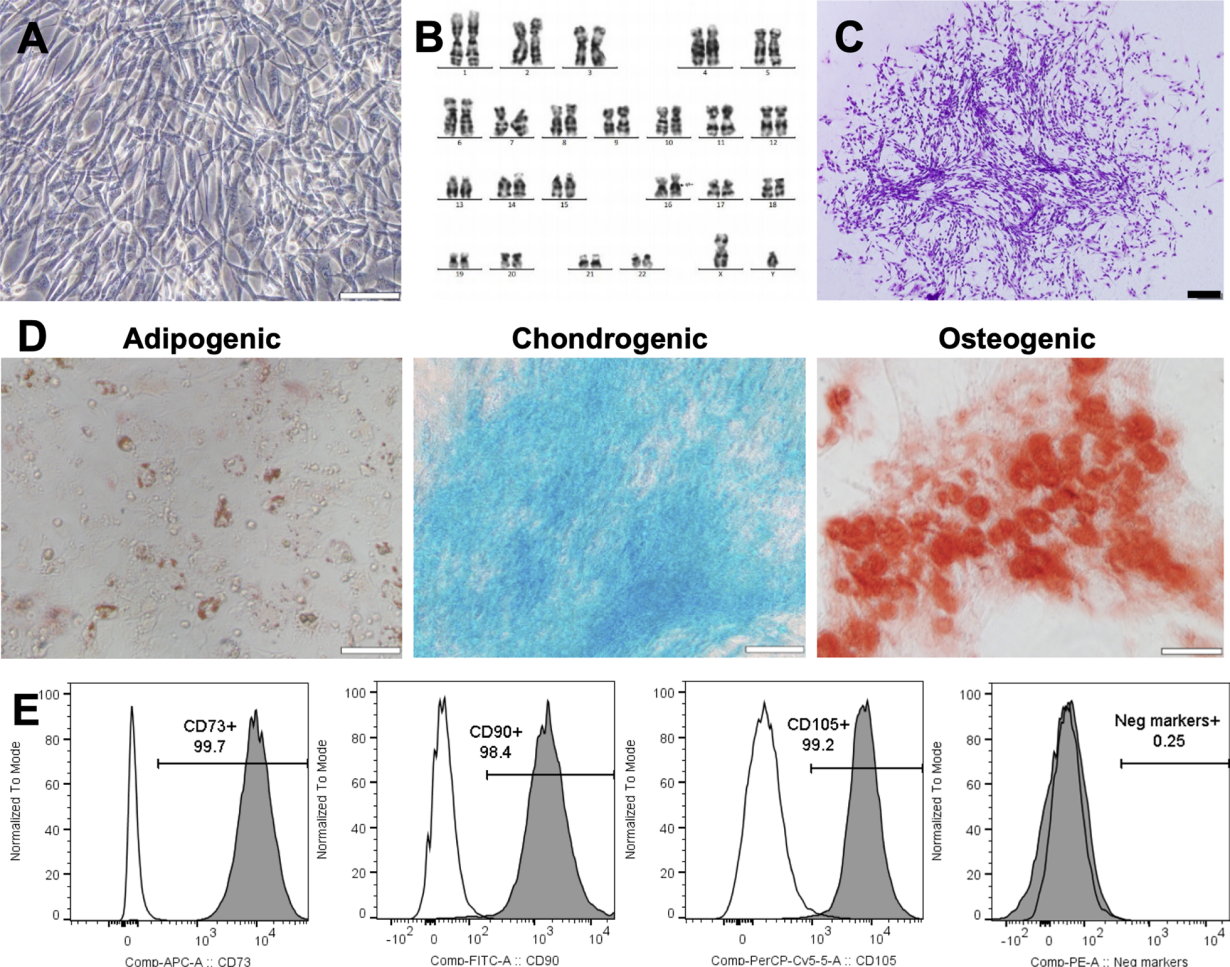
Characterization of hUC-MSC sources for allogeneic administration to patients with severe BPD. **a** hUC-MSCs were obtained from a healthy donor after written informed consent was given. The morphology of hUC-MSCs (P3) expanded in xeno- and serum-free culture medium was spindle-shaped, and the cells were adherent, forming a monolayer in 2D culture. **b** The cells maintained a normal karyotype after 6 passages in culture in vitro, with a population doubling time of 28 ± 1.3 h. **c** The hUC-MSCs exhibited colony-forming features (140 ± 15 CFU/1000 cells, mean ± SEM, n = 3) and **d** were able to differentiate into three lineages. **e** Assessment of MSC markers using flow cytometry confirmed the expression of MSC-positive markers (CD73, CD90, and CD105 > 98%) and less than 2% negative markers. *Scale bar: 100 µm*

Our data showed that the UC-MSC line exhibited plastic adherent properties and a spindle- and fibroblast-like morphology (Fig. 1a), with a population doubling time of 24 ± 0.6 h (n = 3, mean ± SEM). Propagation of UC-MSCs up to passage 6 introduced no karyotypical abnormalities, and the cells maintained a normal 46XY karyotype as indicated by the G-banding technique (Fig. 1b). These cells were also able to form 519 ± 80 CFU/1000 cells (mean ± SEM, n = 3) (Fig. 1c). Further analysis of the differentiation potential confirmed that the UC-MSC line could undergo adipogenic, chondrogenic, and osteogenic differentiation processes, illustrated by positive staining with Oil O Red, Alcian Blue, and Alizarin Red, respectively (Fig. 1d). Analysis of the expression patterns of positive markers, including CD73, CD90, and CD105, showed that more than 99% of the cells expressed all these markers, and less than 2% expressed negative markers, including CD11b, CD19, CD34, CD45, and HLR-DR (Fig. 1e). These results fulfilled the minimum criteria for MSCs proposed by the International Society for Cellular Therapy (ISCT, Table 2).

**Table 2:** Release criteria of allo-UC-MSC administration

Transplantation	Patient 1		Patient 2		Patient 3		Patient 4	
	1st	2nd	1st	2nd	1st	2nd	1st	2nd
Cell doses (× 10^6^ cells/kg)	1.0	1.0	1.0	1.0	1.0	1.0	1.0	1.0
Cell viability (%)	97	97	96	98	98	98	97	89
CD73 (%)	97.9	98.5	98.5	98.3	98.4	99.63	99.5	99.6
CD90 (%)	100	100	100	100	100	99.7	100	99.9
CD105 (%)	100	100	100	100	100	100	100	100
Negative markers* (%)	0.9	0.3	0.3	0.7	0.1	0.1	0.0	0.0
Microorganism and fungal tests	Negative							
Mycoplasma	Negative							
Endotoxin (EU/ml)	< 0.1	< 0.05	< 0.1	< 0.1	0.073	< 0.05	< 0.05	< 0.05
Karyotyping	46, XY, 16qh +							
CFU assay (CFU per 1000 cells)	519 ± 80							
Adipogenesis	Pass							
Chondrogenesis	Pass							
Osteogenesis	Pass							

^*^ CD11b, CD19, CD34, CD45, and HLR-DR

### Patient outcomes as a case report

#### Patient 1

**Fig. 2:**
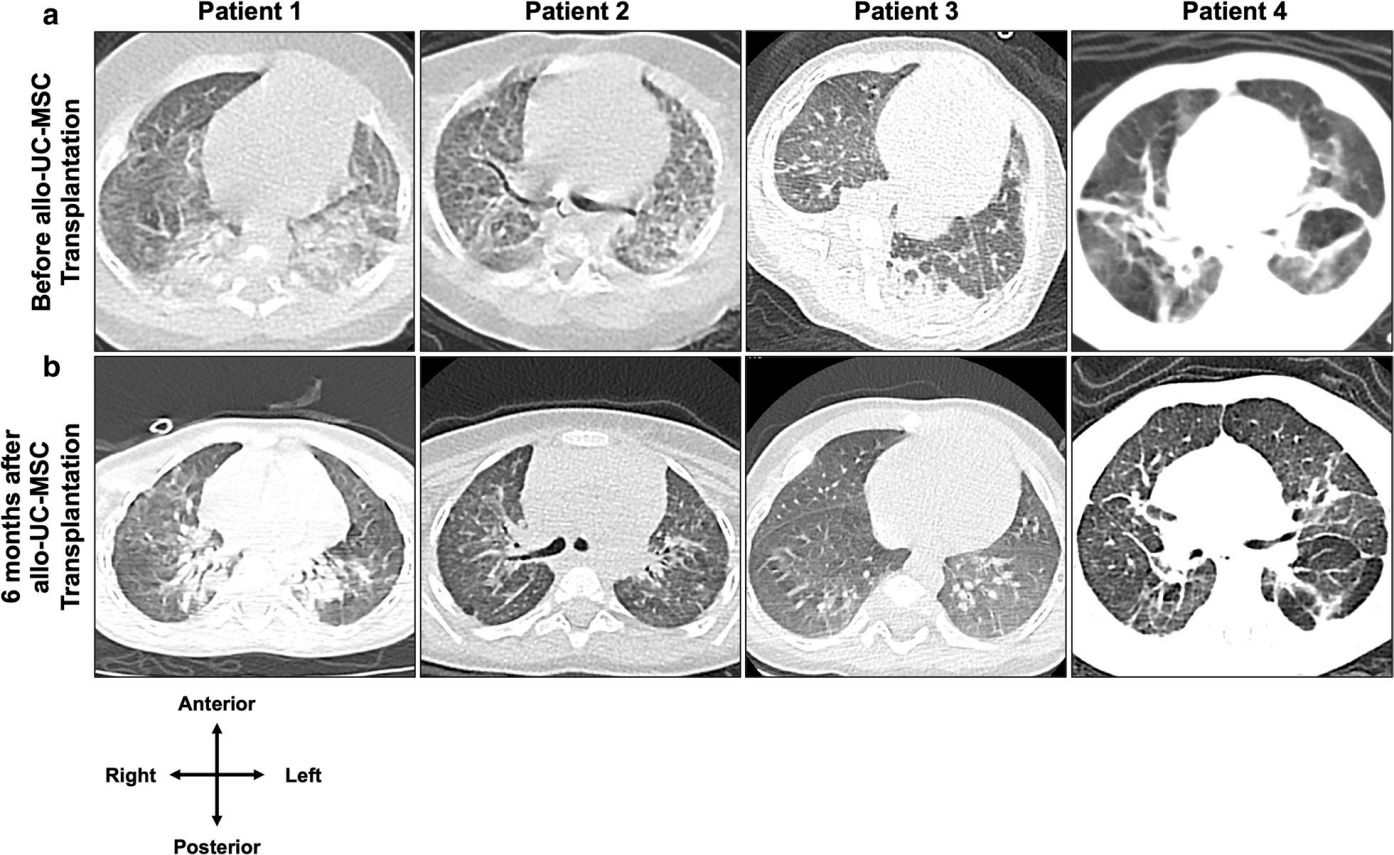
Chest CT scan indicating the recovery progression in lung structure before (**a**) and after (**b**) UC-MSC administration in all four infants with BPD

**Fig. 3:**
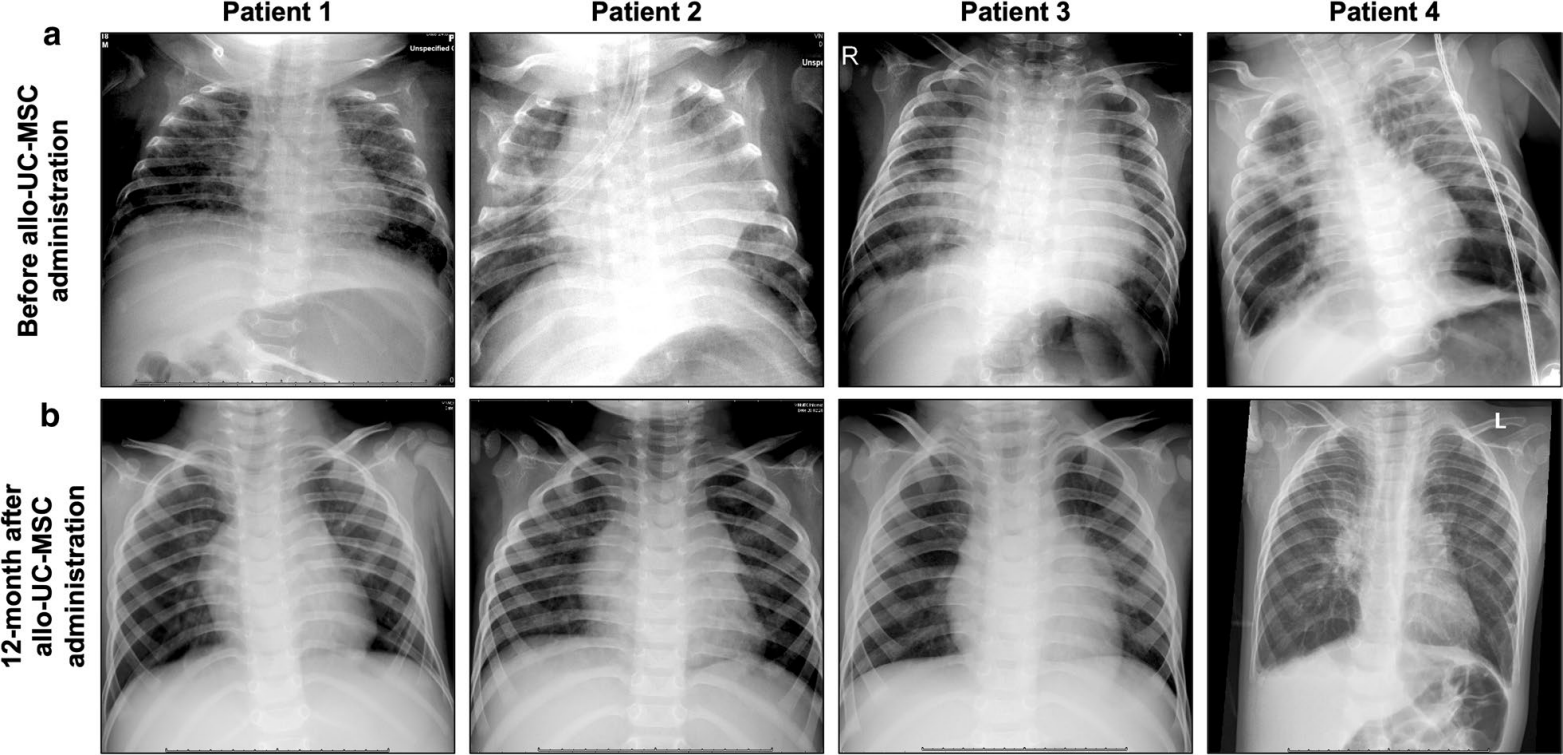
Chest radiographs of the four patients enrolled in the study showing the changes in cystic fibrosis before (**a**) and 12 months after administration (**b**). The results indicate the progressive recovery of the lungs, with more air entering both lungs and a reduction in fibrosis after allo-UC-MSC administration

An extremely premature girl (24 weeks and 5 days, first born of twins) was born by C-section due to premature rupture of the placental membrane with a body weight (BW) of 720 g. Soon after birth, the patient developed signs of respiratory distress syndrome with retraction followed by apnea, cyanosis (SpO_2_ ranged from 60 to 70%) and bradycardia with a heart rate below 100 bpm. She was immediately intubated and placed on mechanical ventilation, with a peak inspiratory pressure (PIP) of 18 cmH_2_O and a positive end-expiratory pressure (PEEP) of 5 cmH_2_O. Chest X-ray showed a stage 2 hyaline membrane requiring one dose of surfactant (Curosurf) at 200 mg/kg BW. Heart ultrasound detected patent ductus arteriosus (PDA), which closed after one course of paracetamol (15 mg/kg/6 h) for seven days, and no evidence of pulmonary artery hypertension (PAH) was observed on echocardiogram after treatment. In addition, the patient suffered from septicemia caused by *Staphylococcus epidermidis*, resulting in necrosis at the distal phalanx of the left little and ring fingers and requiring antibiotic treatment. In the first 2 months, the patient was supported with synchronized intermittent mandatory ventilation (SIMV); the patient was then switched to continuous positive airway pressure therapy (CPAP) at 7 cmH_2_O and 50% FiO_2_ for the following 1.5 months. At 3.5 months postnatal age, the patient was diagnosed with BPD and continued to receive oxygen support via a nasal cannula at 0.5–1 L/min. Nebulized corticosteroids at 100 mcg/kg 4 times/day were administered for a 1-month period. A combination of diuretics (furosemide at 1 mg/kg/12 h), spironolactone (2 mg/kg/12 h) and bronchodilators (inhaled β2-agonists) together with nutrient enhancement (high-calorie nutrition and supplementation with vitamins E, A, K) were initiated for 2 months. However, at 4.5 months postnatal age, the patient’s BPD was not improved, with the SpO_2_ off oxygen support dropping to 90%. The chest CT scan and X-ray at 3.5 months postnatal age confirmed the formation of diffuse fibrosis, atelectasis in the upper lobes of both lungs and significant air trapping in both lower lobes (Figs. 2a, 3a).

Before allo-UC-MSC administration, chronic hypercapnia was confirmed by ABG analysis with the following measured values: pH of 7.31, PaCO_2_ of 68 mmHg, HCO_3_– of 41.3 mmol/L, and PaO_2_ of 73 mmHg. PAH was determined based on a maximum pulmonary artery pressure (PAP) of 40 mmHg and illustrated on echocardiogram, and the pro-BNP level was high (1942 ng/mL). Oral sidenafil (1.5 mg/kg/6 h) and bosentan (1 mg/kg/8 h) were administered when the patient was 4 months old. The allo-UC-MSC administration was performed at 144 days postnatal age (47 weeks gestational age). No signs of serious adverse events were observed during the two interventions. Three days after the second infusion, the patient could breathe spontaneously with an SpO_2_ of 96% without oxygen support. The patient was discharged at 161 days postnatal age (17 days postadministration).

At the first follow-up visit, the patient was alert, had a BW of 4 kg and was spontaneously breathing, with an SpO_2_ of 96% without oxygen support. Blood gas analysis revealed a reduction in the saturated CO_2_ in the blood as follows: pH of 7.5, PaCO_2_ of 33.6 mmHg, HCO_3_- of 26.9 mmol/L, BE of 4 mmol/l and PaO_2_ of 46 mmHg. The pro-BNP level had dropped to 351.9 ng/ml, leading to the termination of PAH treatment at 4 months postadministration. At the 1-month follow-up examination, the patient was conscious and active, and her BW had increased to 4.3 kg, with air fully entering both lungs. She spontaneously breathed and had an SpO_2_ of 97% on room air without oxygen support. The laboratory tests revealed that her pH (7.37), PaCO_2_ (46.3 mmHg), HCO_3_- (27 mmol/L), and PaO_2_ (42 mmHg) remained stable post administration. Hematological analysis also confirmed the absence of inflammation and sepsis, as indicated by the Hgb level (129 G/L), white blood cell count (WBC, 6.1 G/L), and neutrophil level (6.4%). At the 6-month visit, the patient no longer required oxygen support, with her SpO_2_ reaching 100%, and she exhibited good air entry into the lungs, no sign of dyspnea and ABG results in the normal ranges (pH: 7.37, PaCO_2_: 38 mmHg, HCO_3_–: 21.9 mmol/L, PaO_2_: 41 mmHg, and SpO_2_: 100%); the pro-BNP level was 283.1 ng/ml. A reduction in lung fibrosis was observed on chest CT without PAH (Fig. 2b). At 12 months postdischarge, the patient’s condition was improved. Her BW had increased to 7.5 kg. ABG results were in the normal ranges (pH: 7.34, PaCO_2_: 35.5 mmHg, PaO_2_: 87 mmHg, BE: − 6; HCO_3_–: 19.3 mmol/l), and her pro-BNP level was 154.2 without PAH treatment. Lung fibrosis was reduced on chest X-ray at the 12-month visit (Fig. 3b). The detailed progression of the patient’s condition is described in Additional file 1: Table S1.

#### Patient 2

The second premature neonate was the twin of patient 1 and was enrolled in this study with a BW of 650 g. Similar to her twin sister, the patient suffered from respiratory distress syndrome, including gasping, followed by apnea, bradycardia, and cyanosis with an SpO_2_ between 50 and 60%. The patient was positive pressure-ventilated by a bag mask and then intubated and placed on a ventilator in SIMV mode (with ventilator parameters similar to those in the first case). Chest X-ray revealed a stage 3 hyaline membrane, and a surfactant was given at a dose of 200 mg/kg BW on the 1st and 3rd days after birth. A large PDA was detected by cardiac echography, requiring one course of indomethacin (0.2 mg/kg/12 h) within the first postnatal week as previously described [22]. After 2 months on SIMV, ventilation support was switched to CPAP with a PEEP of 6 cmH_2_O and 40% FiO_2_. After treatment, the PDA size was reduced (1 mm), and the shunt size was small; however, the size increased gradually and reached 3.6 mm at 3 months. The PDA was maintained at a large size and required surgical closure at 3.5 months of age. Although the PDA was closed without complications, PAH was observed (38 mmHg), and the pro-BNP level was 2223 ng/ml, leading to treatment with 1 mg/kg/6 h sildenafil and 2 mg/kg/8 h bosentan. After the operation, the patient was on CPAP at 6 cmH_2_O at an FiO_2_ of 30% before switching to nasal cannula oxygen at a rate of 1 L/min after 1 month to maintain a stable SpO_2_ between 93 and 97%. X-rays and chest CT scans at 4 months of age indicated diffuse fibrosis in the lung structures, with atelectasis in the upper lobes of both lungs and significant air trapping in both lower lobes (Figs. 2a and 3a). The patient was confirmed as having BPD and treated with nebulized corticosteroids (100 mcg/kg 4 times/day), diuretics (furosemide, 1 mg/kg/12 h), spironolactone (2 mg/kg/12 h), bronchodilators (inhaled β2-agonists) in combination with ipratropium bromide and other supportive measures (high-calorie nutrition, vitamins E and A, etc.) for 6 weeks. However, the patient’s condition did not improve, and she remained dependent on oxygen support, leading to allo-UC-MSC administration at 151 days postnatal age. Prior to intervention, ABG analysis revealed a pH of 7.6, PaCO_2_ of 37.9 mmHg, PaO_2_ of 35 mmHg, increased HCO_3_– of 29.1 mmol/L and BE of 8 mmol/L. Three days after administration, the patient was discharged with oxygen support via nasal cannula at 1 L/minute, a respiration rate of 64–67 breaths/minute, and an SpO_2_ of 83% (FiO_2_: 21%).

At the first follow-up visit, the patient’s body weight had increased to 4.3 kg, her heart rate was 145 bpm, and she still required oxygen support at 0.5 l/min to maintain an SpO2 over 92% (83% without oxygen support). The ABG results showed a PaCO_2_ of 67 mmHg, HCO_3_– of 32.3 mmol/L, PaO_2_ of 36 mmHg, and BE of 6 mmol/l. The total hemoglobin level, WBC level, and neutrophil percentage were 129, 6.1 G/L, and 6.4%, respectively. The patient’s condition had improved by her 1-month follow-up visit, with reductions in both herPaCO_2_ and HCO_3_–, while her SpO_2_ was maintained at 94–98% on oxygen via a cannula at 0.5 l/min. Two months after the first hUC-MSC administration, home oxygen monitoring results confirmed that the patient could breathe normally, and her SpO_2_ had reached 95% (Additional file 3: Table S3).

From the 6-month follow-up onwards, the patient’s health had stabilized under normal conditions, with her BW reaching 8 kg at the 12-month visit. All ABG tests were within normal parameters at the 6-month visit, further confirming the positive changes of the patient from BPD. Her SpO_2_ was maintained at 95% at the 6-month visit and reached 100% at the 12-month follow-up. Blood gas analysis at 12 months showed that all parameters were within the normal limits without oxygen support. The chest CT scan at the 6-month visit revealed a reduction in lung fibrosis (Fig. 2b). A normal chest X-ray was observed at the 12-month follow-up (Fig. 3b). It is important to note that the maximal PAP recorded at the 6-month visit was 46 mmHg, with a pro-BNP level of 511 ng/ml, leading to the administration of sildenafil (1 mg/kg/12 h). At the 12-month visit, the maximum PAP was 37 mmHg, and the pro-BNP level was reduced to 202 ng/mL; therefore, a lower dose of sildenafil (0.5 mg/kg/12 h) was given. The detailed progression of the patient’s condition is described in Additional file 2: Table S2.

#### Patient 3

A 34-week-old male infant was prematurely born due to premature rupture of the placental membrane and had a BW of 2.4 kg at birth. The patient was diagnosed with hyaline membrane disease and required ventilator support. After 3 consecutive treatments with a surfactant, he was successfully weaned off of mechanical ventilation at 3 months postnatal age. However, he still depended on oxygen support at a rate of 1 L/min via a sponge cannula. The diagnosis of BPD with vocal cord scarring and laryngomalacia combined with periventricular leukomalacia was confirmed using nasopharyngoscopy, CT and MRI.

Upon admission to Vinmec International Hospital, the patient was supported with oxygen at a rate of 1 L/min via nasal cannula to maintain the target SpO_2_ above 92%. The SpO_2_ dropped dramatically to 60% without oxygen support or crying. The patient suffered from severe chronic hypercapnia with pH, BE, PaCO_2_ and HCO_3_– levels maintained at 7.35, 12 mmol/L, 63.6 mmHg and 67.2 mmol/L, respectively, whereas his SpO_2_ and PaO_2_ were relatively low (60% at an FiO_2_ of 21% and 44 mmHg, respectively). No cardiovascular malfunction or PAH was detected on echocardiogram, and the pro-BNP level was 176.5 ng/ml. The patient was diagnosed with CMV infection, with a viral load of 1.44 × 10^5^ copies/ml in the endotracheal fluid. After completion of CMV treatment with valganciclovir for 3 weeks, the chest CT scan and radiograph revealed lung fibrosis with significant air trapping in both lungs and lung inflammation (Figs. 2a and 3a), and the patient could not be weaned off of oxygen. He was dependent on oxygen at a rate of 1 L/min via nasal cannula to maintain an SpO_2_ between 94–96%.

Before administration, the patient still suffered from chronic hypercapnia with the following parameters: pH 7.51, PaCO_2_ 59 mmHg, HCO_3_– 47.2 mmol/L, and PaO2 57 mmHg. He required oxygen support via nasal cannula at 1 L/min to maintain an SpO_2_ between 92–97%; without oxygen support, his SpO_2_ was as low as 70% (FiO_2_: 21%). The PCR results and hematological analysis (WBC: 23.9 G/L, neutrophils: 20.9%, and Hgb: 95 G/L) confirmed that the patient no longer carried CMV; he did not suffer from inflammation, nor did he have sepsis. Allo-UC-MSC administration was performed at 173 days postnatal age with no signs of severe adverse events. The patient was discharged 13 days after the first intervention with oxygen support via nasal cannula at 0.5 L/min with an SpO_2_ ranging between 93 and 98%.

At the first visit, the patient’s general condition was fair, and he was conscious, with his BW slightly increased to 5.3 kg. He was still receiving oxygen at 0.5 l/min via cannula to maintain an SpO_2_ at 92–98%. The patient’s hypercapnic condition was reduced, with the following ABG test results: pH of 7.46, PaO_2_ of 45 mmHg, PaCO_2_ of 52.6 mmHg and HCO_3_– of 38 mmol/L. His SpO_2_ without oxygen support had increased to 85% on room air. The total blood count results remained in the normal ranges. The blood C-reactive protein (CRP) level was 0.2 mg/L, confirming that the patient had not developed an inflammatory response. At the 1-month follow-up, the patient was still dependent on oxygen support at a rate of 0.5 L/min to maintain an SpO_2_ level between 95% and 98. At two months postadministration, the patient was independent of active oxygen support, with an SpO_2_ of 96–98%.

The clinical team observed the progression in patients’ condition at the 6-month visit. The patient was conscious and was able to crawl, laugh, and actively respond to his parent’s voice. Due to the complication of periventricular leukomalacia, an additional Denver II test was conducted at the 6-month examination, and the results confirmed that the patient’s gross motor function was similar to that expected at 3 months, his language ability was equivalent to that expected at 5–6 months, his fine motor adaptive skills were equivalent to those expected at 3 months, and his personal-social skills were equivalent to those expected at 5 months. Moreover, improved respiratory function was also documented, with better airflow in both lungs, no crackles or rales, and no signs of retraction or nasal flaring at the 6-month visit. All ABG results remained stable at the 12-month visit, with no sign of respiratory distress syndrome, an improved saturated oxygen level (SpO_2_: 100%) and a normal CO_2_ level in the blood (pH of 7.4, PaO_2_ of 72 mmHg, PaCO_2_ of 34.8 mmHg, HCO_3_– of 21.5 mmol/L; BE of − 3 mmol/l). Investigation of the patient’s lungs with CT at the 6-month visit indicated a reduction in fibrosis and the gradual recovery of lung function. Chest X-rays at the 12-month visit further confirmed the progression (Fig. 3b). The detailed progression of the patient’s condition is described in Additional file 3: Table S3.

#### Patient 4

A premature female infant was born at another hospital at 28 weeks gestation due to premature rupture of the placental membranes with a birth weight of 1400 g. She rapidly developed respiratory distress syndrome and required mechanical ventilation. A single dose of surfactant was given (100–200 mg/kg) on the first day. After that, the patient was placed on CPAP for a month, followed by oxygen support at 0.5–1 L/min until she reached 36 weeks old. Dexamethasone treatment using the Dexamethasone: A Randomized Trial (DART) protocol was advised for one week to further improve the patient’s condition. The patient was successfully weaned from oxygen support and discharged at 37 weeks with an SpO_2_ ranging between 93 and 95%. However, 2 days postdischarge, the patient developed dyspnea with acute respiratory distress and returned to the hospital, where she stayed for the next 2 months.

The patient was referred to Vinmec Hospital at 4 months old with malnutrition (BW of 3 kg). Although oxygen support was maintained at 1 L/min via nasal cannula, her SpO_2_ was relatively low (80%). Auscultation showed poor air entry into the lungs with crackles and rales. Her heart rate was high (200–220 bpm), with evident cyanosis and an SpO_2_ of 80 on 24% oxygen. The patient was intubated immediately and placed on a ventilator in SIMV mode (PIP at 23 cm H_2_O, PEEP at 5.5 cm H_2_O, and FiO_2_ at 50%). Five days after the treatment, ventilation support was switched to sponge cannula with oxygen flowing at 1 L/min. The ABG examinations revealed the following: pH of 7.49, PaCO_2_ of 38.6 mmHg, HCO_3_– of 29.5 mmol/l, and PaO_2_ of 60 mmHg with FiO2: 40%. Furthermore, a complete blood count showed a low platelet count (53 G/L), while the WBC, neutrophil, and Hgb results were 5.8, 1.3 and 112 G/L, respectively. An echocardiogram was performed when the patient was stable and showed a pressure gradient through the tricuspid valve at 28 mmHg. The pro-BNP level was 8065 pg/ml. Hence, the patient was treated with 0.5 mg/kg/8 h sildenafil. The viral tests confirmed a CMV infection (460 copies/ml), which was treated with valganciclovir for 21 days. The results of a chest X-ray and CT scan indicated severe lung fibrosis and substantial air trapping in both lungs (Figs. 2a and 3a).

Two UC-MSC administrations were carried out without adverse events when the patient was 160 days old. Four days after the first administration, the patient could breathe spontaneously at 55–62 breaths/minute. On the day of discharge (a week after the second administration), the patient breathed spontaneously with an SpO2 of 95% without oxygen support.

At the 7-day examination, the patient still suffered from dyspnea, with a respiration rate of 53 breaths/minutes. An increase in the SpO_2_ level to 95% was also recorded. The pro-BNP level was reduced to 136.7 ng/ml. Hematological analysis confirmed that no sepsis or inflammatory reaction had occurred after MSC administration, with a WBC count of 9.8 G/L, neutrophil percentage of 12.1%, Hgb level of 112 G/L and platelet count of 61 G/L. One month postdischarge, the patient was conscious and active, with a BW of 4 kg. The SpO_2_ was increased to 98% without oxygen support, suggesting that the patient’s respiratory function had recovered.

At the 6-month visit, respiratory distress was assessed as mild. The SpO_2_ had stabilized at 97%. The 12-month follow-up corroborated the conclusion that the patient had recovered from BPD, with a normal SpO_2_ of 97%, pH of 7.34, PaCO_2_ of 39.8 mmHg, HCO_3_– of 20.8 mmol/l, BE of − 4 mmol/l, and PaO_2_ of 73 mmHg. At 12 months after intervention, it is worth mentioning that the patient had recovered well with regard to both her SpO_2_ and PaO_2_ levels, which were 100% and 72 mmHg, respectively. Evaluation of the lung structure on CT scans demonstrated that the fibrotic area was reduced (Fig. 2b), while alveolation and maturation of the lung had become obvious. Further assessment of the lung structure using chest X-rays at the 12-month follow-up showed no signs of atelectasis or hyperexpansion in either lung (Fig. 3b). The detailed progression of the patient’s condition is described in Additional file 4: Table S4.

## Discussion

All four patients tolerated the allo-UC-MSC infusion well, and no prespecified infusion-related adverse events were recorded after either the first or second administration. Specifically, no significant changes in heart rate, mean arterial pressure, oxygen saturation or body temperature were observed in any of the four infants (Additional file 5: Figure S1). These results, together with the detailed hematological analysis reported in each case, confirmed that allo-UC-MSC administration does not trigger any complications during or 72 h after infusion. A previous study reported the safety outcomes of the allogeneic administration of human UCB-MSCs, in which nine preterm infants received either a single dose of 1 × 10^7^ cells/kg or 2 × 10^7^ cells/kg [23]. In another single-center, open-label phase 1 trial, Lim’s group administered 1 × 10^6^ human amnion epithelial cells to six preterm infants with established BPD and reported the safety profile at 2 years postadministration [24, 25]. In these trials, as in ours, no infusion toxicity or allogeneic UC-MSC intervention-associated adverse events were recorded. These results were also supported by a preclinical study evaluating the long-term safety of the allogeneic administration of MSCs in a rodent model of BPD, which reported no adverse lung effects postadministration together with persistent recovery in respiratory function and lung condition [26].

Identifying the appropriate cell source plays an important role in the success of therapy. In our study, UC-MSCs were isolated from a single healthy donor as an allogeneic source for MSC administration due to the following reasons: (1) collection of UC samples is a noninvasive process, as the UC is medical waste discarded at birth, eliminating the need for bone marrow aspiration from infants with established BPD; (2) their rapid proliferation capacity (24 ± 0.6 h); and (3) their maintenance of a normal karyotype and differentiation potential during in vitro culture. Moreover, UC-MSCs have a higher paracrine potency than adult tissue-derived MSCs [27]. Moreover, previous studies performed using human amnion epithelial cells and UCB-MSCs suggest that MSCs derived from perinatal sources might be the optimal cell source for future clinical treatments of infants with BPD [23, 24].

In terms of the transplantation route, our preliminary data demonstrate the safety profile of allo-UC-MSC administration via IV infusion in neonates with established BPD. During the follow-up period, all patients exhibited the progressive recovery in lung function and stopped being dependent on mechanical oxygen support as early as 4 days after the first administration and as late as 2 months postadministration. In preclinical studies, three main routes have been tested in the treatment of BPD: intraperitoneal (usually conducted in animal models but not translatable to the clinical setting), IV, and intranasal or intratracheal administration [28]. However, the potential effects of therapies administered via these routes are contradictory. In hypoxia-exposed mice, human UC-MSCs delivered via the intraperitoneal route showed improved lung function, while those administered the cells via the intratracheal route did not [29]. Furthermore, a systematic analysis of preclinical studies suggested that MSCs administered intravenously had better effects than those administered via the intratracheal route [30]. In contrast, local intratracheal delivery of MSCs was reported to be more effective and to better attenuate hyperoxia-induced lung damage than systemic IV administration [19]. Hence, future studies are needed to confirm the suitable administration route of MSC therapy for BPD infants.

To achieve optimal therapeutic effects, determination of the administration timing is a critical issue that remains to be solved. In our study, all four patients were diagnosed with BPD and underwent traditional medicaments without improvements in their conditions prior to allo-UC-MSC administration. Previous studies have shown the safety profile of MSCs in the prevention of BPD and acute lung injury in infants at risk [20, 23, 31]. A recent study reported the safety of allogeneic administration of BM-MSCs in two severe and advanced BPD infants and suggested that MSC therapy should be conducted in early stages of the disease [32]. Because the respiratory outcomes of premature infants can vary widely and no specific early marker of BPD exists, the prediction of BPD development can be challenging. The earliest time at which BPD can be predicted is day 4 of life [33]. To confirm the diagnosis of BPD, subsequent studies found that BPD patterns typical of lung diseases emerge during the first 14 days of life. Moreover, during this period, several risk factors could affect disease severity, including late surfactant deficiency, sepsis, inflammation, and PDA [34, 35]. Furthermore, 50% of infants with pulmonary deterioration and nearly 70% of infants with early persistent pulmonary deterioration reportedly develop BPD [36]. Hence, it is important to determine the timing at which MSC therapy could be administered as a preventive treatment (intervention conducted within the first 14 postnatal days) or for BPD treatment (infants confirmed to have BPD).

In terms of study limitations, first and foremost, we can only report our safety data and cannot draw conclusions about either the efficacy or long-term safety of allo-UC-MSCs for the treatment of BPD because only four patients were included and no control group was utilized. Based on the safety profiles of UC-MSCs in the treatment of BPD, it is important to perform a randomized, placebo-controlled phase 2 clinical trial with a larger cohort and a primary focus on safety and secondary outcomes, including respiratory, inflammatory, systemic, and MSC biological endpoints.

## Conclusions

Our current study provides the evidence of the safety of the allogeneic administration of hUC-MSCs in patients with established BPD. The results also showed that BPD infants were weaned off of oxygen support with reduction of lung fibrosis and progression of lung function post-administration. A study with a larger cohort and a control group is needed to draw more accurate conclusions.

## Supplementary information

**Additional file 1: Table S1.** Clinical data and detailed examinations of Patient 1.

**Additional file 2: Table S2.** Clinical data and detailed examinations of Patient 2.

**Additional file 3: Table S3.** Clinical data and detailed examinations of Patient 3.

**Additional file 4: Table S4.** Clinical data and detailed examinations of Patient 4. NI: No information, unable to obtain samples from patient.

**Additional file 5. Figure S1:** Hemodynamic and respiratory functions during 72h of UC-MSC infusion. Mean (±SEM) values of four patients for (a) heart rate (beats per minute), (b) temperature (°C), and (c) arterial oxygen saturation as measured by pulse oximeter (SpO_2_; %) at base line and every 3 hours from start of UC-MSC infusion up to 72h post-transplantation. 

## Data Availability

The authors declare that [the/all other] data supporting the findings of this study are available within the article [and its additional files].

## Abbreviations

allo-UC-MSCs: Allogeneic administration of umbilical cord-derived mesenchymal stem/stromal cells
ABG: Arterial blood gas analysis
BE: Base excess
BPD: Bronchopulmonary dysplasia
BW: Body weight
HCO_3_-: Bicarbonate
ITP: Idiopathic thrombocytopenia purpura
PaCO_2_: Partial pressure of carbon dioxide
PAH: Pulmonary artery hypertension
PaO_2_: Partial pressure of oxygen
PAP: Pulmonary artery pressure
PBW: Patient body weight
PEEP: Positive end-expiratory pressure
PIP: Peak inspiratory pressure
SpO_2_: Saturated oxygen level in blood
UC-MSCs: Umbilical cord-derived mesenchymal stem/stromal cells
