# Supplementary material for: Allogeneic administration of human umbilical cord-derived mesenchymal stem/stromal cells for bronchopulmonary dysplasia: preliminary outcomes in four Vietnamese infants
Source: J Transl Med. 2020 Oct 20;18:398. doi: 10.1186/s12967-020-02568-6 (PMC7576694; doi:10.1186/s12967-020-02568-6)
Supplement: Supplementary file 1 — Additional file 1: Table S1. Clinical data and detailed examinations of Patient 1. [file 12967_2020_2568_MOESM1_ESM.docx]

**Table S1: Clinical data and detailed examinations of Patient 1.**

| Tests | Parameters | At birth | Prior to  allo-UC-MSC administration | After allo-UC-MSC administration (Discharged) | | | |
| --- | --- | --- | --- | --- | --- | --- | --- |
|  |  |  |  | **7 days** | **1 month** | **6 months** | **12 months** |
| Patient condition | *Body weight (kg)* | 0.72 | 3.6 | 4 | 4.3 | 6 | 7.5 |
|  | *Heart rate (bpm)* | 145 | 160 | 147 | 152 | 125 | 115 |
| Arterial blood gas (ABG) | *pH* | 7.2 | 7.31 | 7.4 | 7.37 | 7.37 | 7.34 |
|  | *BE (mmol/L)* | -3 | 16 | 7 | 2 | -3 | -6 |
|  | *PaCO_2_ (mmHg)* | 61.7 | 68 | 49.9 | 46.3 | 38 | 35.5 |
|  | *HCO_3_- (mmol/L)* | 24.5 | 41.3 | 31.8 | 27 | 21.9 | 19.3 |
|  | *PaO_2_ (mmHg)* | 83 | 73 | 46 | 42 | 41 | 87 |
|  | *SpO_2_ (%)* | 90 | 91 | 96 | 97 | 100 | 100 |
| Total blood count analysis | *WBC (G/l)* | 12.5 | 11.2 | 15.8 | 6.1 | 7.2 | 11.5 |
|  | *Neu (%)* | 52.2 | 11.9 | 53.5 | 6.4 | 16.3 | 21.3 |
|  | *Lym (%)* | 22.8 | 74.1 | 32.8 | 81.1 | 75.1 | 68.8 |
|  | *Hgb (g/l)* | 164 | 109 | 109 | 129 | 134 | 146 |
|  | *Hct (%)* | 49.7 | 34.3 | 33.8 | 40.6 | 39.1 | 41.9 |
|  | *Plt (G/l)* | 265 | 363 | 392 | 194 | 220 | 310 |
|  | RBC (T/l) | 4.11 | 3.8 | 3.87 | 5.06 | 5.04 | 5.33 |
