# Supplementary material for: Allogeneic administration of human umbilical cord-derived mesenchymal stem/stromal cells for bronchopulmonary dysplasia: preliminary outcomes in four Vietnamese infants
Source: J Transl Med. 2020 Oct 20;18:398. doi: 10.1186/s12967-020-02568-6 (PMC7576694; doi:10.1186/s12967-020-02568-6)
Supplement: Supplementary file 2 — Additional file 2: Table S2. Clinical data and detailed examinations of Patient 2. [file 12967_2020_2568_MOESM2_ESM.docx]

**Table S2: Clinical data and detailed examinations of Patient 2.**

| Tests | Parameters | At birth | Prior to  allo-UC-MSC administration | After allo-UC-MSC administration (Discharged) | | | |
| --- | --- | --- | --- | --- | --- | --- | --- |
|  |  |  |  | **7 days** | **1 month** | **6 months** | **12 months** |
| Patient condition | *Body weight (kg)* | 0.65 | 4 | 4.3 | 4.5 | 5.5 | 8 |
|  | *Heart rate (bpm)* | 135 | 140 | 145 | 154 | 115 | 120 |
| Arterial blood gas (ABG) | *pH* | 7.54 | 7.6 | 7.29 | 7.25 | 7.35 | 7.27 |
|  | *BE (mmol/L)* | -2 | 8 | 34 | -5 | -5 | -6 |
|  | *PaCO_2_ (mmHg)* | 24 | 37.9 | 67 | 48.9 | 32.8 | 45 |
|  | *HCO_3_- (mmol/L)* | 20.8 | 29.1 | 32.3 | 21.8 | 18.3 | 20.7 |
|  | *PaO_2_ (mmHg)* | 59 | 35 | 36 | 31 | 58 | 27 |
|  | *SpO_2_ (%)* | 92 | 75 | 83 | 88 | 95 | 100 |
| Total blood count analysis | *WBC (G/l)* | 7 | 5.7 | 6.1 | 9 | 16.3 | 19.9 |
|  | *Neu (%)* | 42.2 | 21.8 | 6.4 | 14.6 | 13.9 | 33 |
|  | *Lym (%)* | 45.9 | 65.5 | 81.1 | 70.8 | 76 | 55.5 |
|  | *Hgb (g/l)* | 151 | 124 | 129 | 127 | 143 | 134 |
|  | *Hct (%)* | 44.5 | 39.2 | 40.6 | 38.6 | 43.2 | 39.6 |
|  | *Plt (G/l)* | 287 | 233 | 194 | 253 | 302 | 294 |
|  | RBC (T/l) | 3.72 | 4.63 | 5.06 | 5.19 | 5.7 | 5.03 |
