# Supplementary material for: Allogeneic administration of human umbilical cord-derived mesenchymal stem/stromal cells for bronchopulmonary dysplasia: preliminary outcomes in four Vietnamese infants
Source: J Transl Med. 2020 Oct 20;18:398. doi: 10.1186/s12967-020-02568-6 (PMC7576694; doi:10.1186/s12967-020-02568-6)
Supplement: Supplementary file 3 — Additional file 3: Table S3. Clinical data and detailed examinations of Patient 3. [file 12967_2020_2568_MOESM3_ESM.docx]

**Table S3: Clinical data and detailed examinations of Patient 3.**

| Tests | Parameters | Admission to Vinmec | Prior to  allo-UC-MSC administration | After allo-UC-MSC administration (Discharged) | | | |
| --- | --- | --- | --- | --- | --- | --- | --- |
|  |  |  |  | **7 days** | **1 month** | **6 months** | **12 months** |
| Patient condition | *Body weight (kg)* | 5 | 5 | 5.3 | 6.2 | 8.5 | 10.6 |
|  | *Heart rate (bpm)* | 157 | 150 | 147 | 145 | 120 | 120 |
| Arterial blood gas (ABG) | *pH* | 7.35 | 7.51 | 7.46 | 7.4 | 7.53 | 7.4 |
|  | *BE (mmol/L)* | 12 | 24 | 14 | 9 | 2 | -3 |
|  | *PaCO_2_ (mmHg)* | 63.6 | 59 | 52.6 | 53.8 | 28.6 | 34.8 |
|  | *HCO_3_- (mmol/l)* | 67.2 | 47.2 | 38 | 33.7 | 24.1 | 21.5 |
|  | *PaO_2_ (mmHg)* | 44 | 57 | 45 | 40 | 82 | 72 |
|  | *SpO_2_ (%)* | 60 | 70 | 85 | 93 | 98 | 100 |
| Total blood count analysis | *WBC (G/l)* | 11.5 | 23.9 | 14.6 | 9.4 | 11.6 | 12.6 |
|  | *Neu (%)* | 46.2 | 20.9 | 60.2 | 35 | 18.5 | 21.4 |
|  | *Lym (%)* | 39.8 | 71.8 | 34.9 | 50.7 | 64.2 | 60.6 |
|  | *Hgb (g/l)* | 103 | 95 | 105 | 117 | 113 | 113 |
|  | *Hct (%)* | 31.2 | 29 | 31.9 | 35.3 | 33.7 | 32.8 |
|  | *Plt (G/l)* | 330 | 334 | 329 | 317 | 139 | 309 |
|  | RBC (T/l) | 3.75 | 3.5 | 3.76 | 4.17 | 4.2 | 4.16 |
