# Supplementary material for: Allogeneic administration of human umbilical cord-derived mesenchymal stem/stromal cells for bronchopulmonary dysplasia: preliminary outcomes in four Vietnamese infants
Source: J Transl Med. 2020 Oct 20;18:398. doi: 10.1186/s12967-020-02568-6 (PMC7576694; doi:10.1186/s12967-020-02568-6)
Supplement: Supplementary file 4 — Additional file 4: Table S4. Clinical data and detailed examinations of Patient 4. NI: No information, unable to obtain samples from patient. [file 12967_2020_2568_MOESM4_ESM.docx]

**Table S4: Clinical data and detailed examinations of Patient 4.** NI: No information, unable to obtain samples from patient.

| Tests | Parameters | Admission to Vinmec | Prior to  allo-UC-MSC administration | After allo-UC-MSC administration (Discharged) | | | |
| --- | --- | --- | --- | --- | --- | --- | --- |
|  |  |  |  | **7 days** | **1 month** | **6 months** | **12 months** |
| Patient condition | *Body weight (kg)* | 3 | 3.8 | 3.9 | 4 | 6.3 | 6.7 |
|  | *Heart rate (bpm)* | 220 | 130 | 130 | 134 | 145 | 148 |
| Arterial blood gas (ABG) | *pH* | 7.49 | 7.5 | 7.38 | NI | 7.34 | 7.48 |
|  | *BE (mmol/L)* | 6 | 2 | 0 | NI | -4 | -1 |
|  | *PaCO_2_ (mmHg)* | 38.6 | 31.9 | 24.2 | NI | 39.8 | 30.2 |
|  | *HCO_3_- (mmol/l)* | 29.5 | 25.1 | 24.9 | NI | 21.5 | 20.8 |
|  | *PaO_2_ (mmHg)* | 60 | 189 | 162 | NI | 39.8 | 87 |
|  | *SpO_2_ (%)* | 80 | 90 | 95 | 98 | 97 | 97 |
| Total blood count analysis | *WBC (G/l)* | 7.3 | 5.8 | 9.8 | 6 | 8.4 | 8.5 |
|  | *Neu (%)* | 28 | 23 | 12.1 | 62.9 | 25.8 | 23.9 |
|  | *Lym (%)* | 57.1 | 70.9 | 75.6 | 33 | 57.3 | 67.9 |
|  | *Hgb (g/l)* | 86 | 112 | 112 | 108 | 117 | 124 |
|  | *Hct (%)* | 26.7 | 34.6 | 42 | 33.7 | 35.8 | 38.5 |
|  | *Plt (G/l)* | 185 | 53 | 61 | 60 | 14 | 246 |
|  | RBC (T/l) | 3.25 | 4.32 | 4.2 | 4.1 | 4.5 | 4.68 |
