# Supplementary figures and images for: Allogeneic administration of human umbilical cord-derived mesenchymal stem/stromal cells for bronchopulmonary dysplasia: preliminary outcomes in four Vietnamese infants
Source: J Transl Med. 2020 Oct 20;18:398. doi: 10.1186/s12967-020-02568-6 (PMC7576694; doi:10.1186/s12967-020-02568-6)

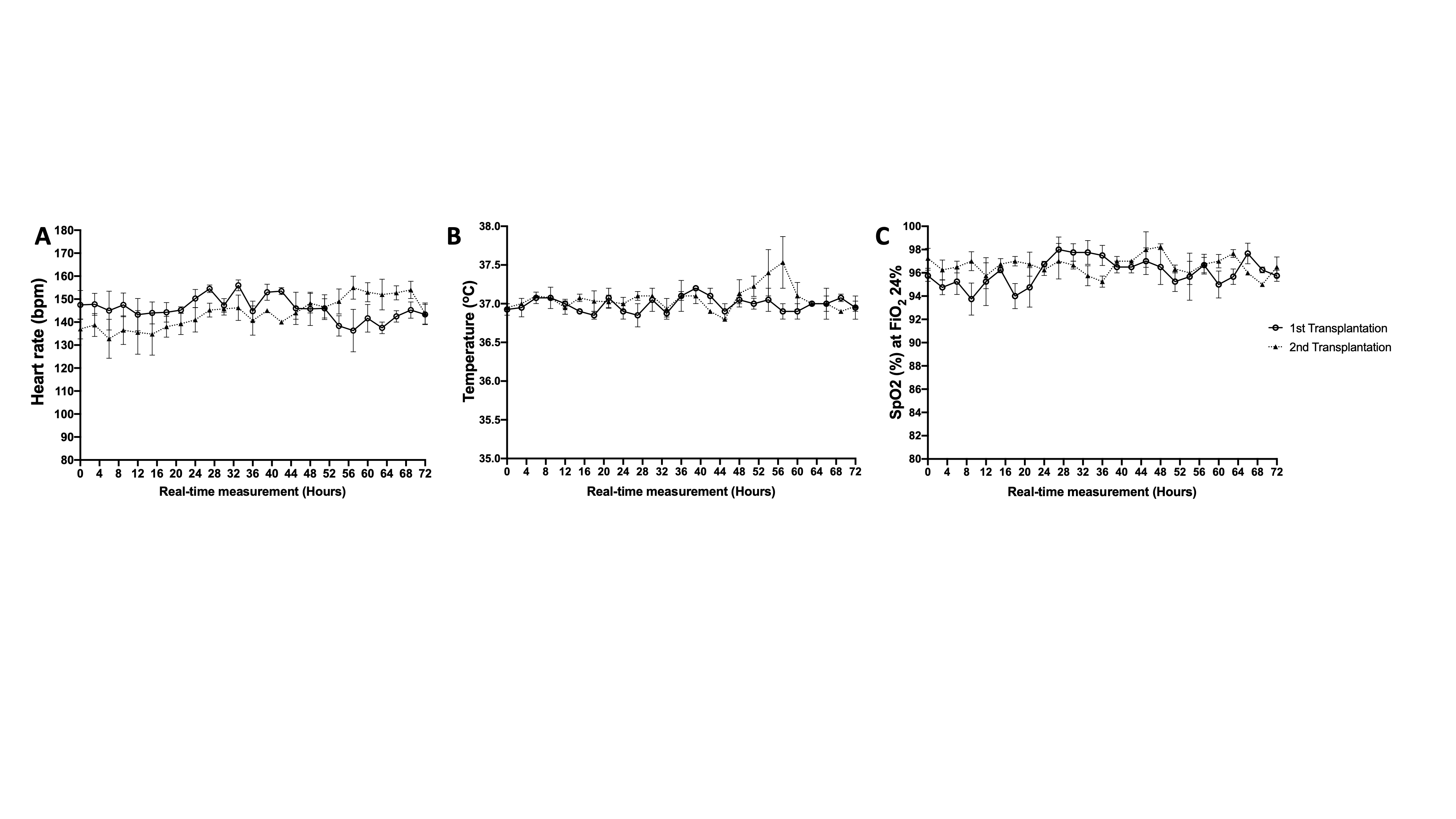

Supplement: Supplementary file 5 — Additional file 5. Figure S1: Hemodynamic and respiratory functions during 72h of UC-MSC infusion. Mean (±SEM) values of four patients for (a) heart rate (beats per minute), (b) temperature (°C), and (c) arterial oxygen saturation as measured by pulse oximeter (SpO2; %) at base line and every 3 hours from start of UC-MSC infusion up to 72h post-transplantation. [file 12967_2020_2568_MOESM5_ESM.docx]
